# An Intelligent Machinery Fault Diagnosis Method Based on GAN and Transfer Learning under Variable Working Conditions

**DOI:** 10.3390/s22239175

**Published:** 2022-11-25

**Authors:** Wangpeng He, Jing Chen, Yue Zhou, Xuan Liu, Binqiang Chen, Baolong Guo

**Affiliations:** 1 School of Aerospace Science and Technology, Xidian University, Xi’an 710071, China; 2 Guangzhou Institute of Technology, Xidian University, Guangzhou 510555, China; 3 School of Aerospace Engineering, Xiamen University, Xiamen 361005, China

**Keywords:** intelligent fault diagnosis, transfer learning, generative adversarial network, domain adaptation

## Abstract

Intelligent fault diagnosis is of great significance to guarantee the safe operation of mechanical equipment. However, the widely used diagnosis models rely on sufficient independent and homogeneously distributed monitoring data to train the model. In practice, the available data of mechanical equipment faults are insufficient and the data distribution varies greatly under different working conditions, which leads to the low accuracy of the trained diagnostic model and restricts it, making it difficult to apply to other working conditions. To address these problems, a novel fault diagnosis method combining a generative adversarial network and transfer learning is proposed in this paper. Dummy samples with similar fault characteristics to the actual engineering monitoring data are generated by the generative adversarial network to expand the dataset. The transfer fault characteristics of monitoring data under different working conditions are extracted by a deep residual network. Domain-adapted regular term constraints are formulated to the training process of the deep residual network to form a deep transfer fault diagnosis model. The bearing fault data are used as the original dataset to validate the effectiveness of the proposed method. The experimental results show that the proposed method can reduce the influence of insufficient original monitoring data and enable the migration of fault diagnosis knowledge under different working conditions.

## 1. Introduction

Intelligent fault diagnosis is used to intelligently identify the health status of mechanical equipment by automatically extracting the hidden fault characteristic information from the monitoring data of the mechanical equipment, which is the current research hotspot in the field of fault diagnosis [1,2,3]. An adaptive bearing fault diagnosis method based on a two-dimensional convolutional neural network (CNN) was proposed by Wang et al., for which maximum retention of the original fault data characteristics benefited from the original signal being used as input [4]. A novel signal-to-image mapping was proposed by Zhao et al., wherein the one-dimensional vibration signal is converted into a two-dimensional gray image and the fault features of the gray image are extracted through the CNN model [5]. A new type of bearing fault diagnosis method was proposed by Xu et al., which combines a deep CNN with random forest (RF) [6]. Chen et al. proposed a new fault diagnosis method based on deep learning, which uses 2D map representations of cyclic spectral coherence (CSCoh) representation and a CNN [7]. Yuan et al. proposed a rolling bearing fault diagnosis method based on a CNN and support vector machine in order to reduce the dependence on manual intervention in the feature extraction process [8]. Yang et al. proposed a novel fuzzy fusion method for fault diagnosis, and the improved CNN model still has good performance even in noisy environments [9].

These studies were mainly based on the two assumptions of machine learning, i.e., the samples have rich label information and are independent and identically distributed [10,11]. However, in actual working conditions, the monitoring data of mechanical equipment have the characteristics of low value density and low availability [12,13]. Yang et al. proposed a new deep learning model to solve the problem of rotating machinery fault diagnosis with small samples [14]. However, under the long-term normal operation of mechanical equipment, the repetition rate of information in the monitored data was high. The data in the normal state accounted for the majority, while the data in the fault state were very few and the typical fault information was missing [15]. Most monitoring data are unlabeled, and labeling data costs a lot of manpower and financial resources, resulting in insufficient data being available for training models. In addition, in practice, the working environment of the equipment is complex and changeable, the monitoring signal is unstable, and some of the collected signals are weak and contain a lot of back-bottom noise [16,17]. The fault characterization changes dynamically, the generalization performance of the classification model trained under the original working conditions is reduced, and it is difficult to apply to the new working conditions [18]. Therefore, the available data in the actual engineering monitoring data are scarce, which makes it difficult to train and obtain an intelligent diagnostic model with high accuracy in identifying the health status of the equipment. Moreover, the diagnostic model trained under a single working condition has low generalization ability, which is trained for each specific working condition. The new diagnostic model still consumes a lot of time, manpower, and financial resources. Researching and exploring advanced technologies and new theories to solve the above problems is a frontier research hotspot in the current intelligent diagnosis of mechanical faults.

Transfer learning (TL) is a new machine learning method that uses existing knowledge to solve problems in different but related fields [19]. TL can solve the learning problem of scarce available data to a certain extent and has been successfully applied in image recognition [20], speech recognition [21], text recognition [22], and other fields. In the field of the intelligent diagnosis of mechanical faults [23], TL has gradually attracted the attention of researchers.

Zhang et al. combined deep CNN with TL to reduce the dependence of the model on training data and accurately identify different fault types [24]. Chen et al. combined multitask learning with TL and proposed a novel model parameter transfer (NMPT) to improve the performance of gear fault diagnosis (GFD) under different operating conditions [25]. Qian et al. proposed a new method for evaluating distribution differences, which is called auto-balancing higher-order Kullback–Leibler (AHKL) divergence; minimizing the difference in domain distribution can be achieved by this method [26]. A deep transfer multiwavelet auto-encoder was proposed by He et al., wherein intelligent fault diagnosis for gearboxes with few training samples was achieved [27]. Zhang et al. proposed a new type of deep transfer diagnosis model based on Wasserstein distance [28]. The learning process of this method is to minimize the Wasserstein distance between the source domain and the target domain by using adversarial training strategies. Zhu et al. proposed a depth domain adaptation method based on two-dimensional CNN to solve this problem, in which vibration signals are converted into images as input samples and domain adaptation is implemented in the last two layers of the network [29]. Zhang et al. trained a domain adaptive CNN model to minimize the maximum mean square error between extracted features; as a result, the features of the source domain and target domain have similar distributions after mapping [30]. The above research results show that the use of existing mechanical fault diagnosis knowledge can identify the health status of mechanical equipment with relevant fault information. However, the effectiveness of the above method is based on certain assumptions: the available data obtained by mechanical equipment under certain working conditions is sufficient, which is inconsistent with the characteristics of monitoring data in actual engineering, and it is difficult to adapt and meet the engineering application requirements of mechanical fault intelligent diagnosis.

A generative adversarial network (GAN) is composed of a generative network and a discriminator network, wherein the generative network and the discriminator network dynamically iterative optimization so that the images generated by the generative network are as close to the real samples as possible [31]. Recently, GAN has also attracted the attention of some researchers. Li et al. proposed a knowledge mapping-based adversarial domain adaptive fault diagnosis method to complete the fault diagnosis analysis under variable work conditions [32]. However, in the actual engineering environment, there is no sufficient available data of machinery faults to train an intelligent diagnostic model. Meanwhile, most of the monitoring data have no label information and the collected data distribution is inconsistent due to the complex and variable working conditions. As a result, the diagnostic model has low accuracy, which makes it difficult to apply to new working conditions. Inspired by GAN and transfer learning, a two-stage fault diagnosis method combining GAN and transfer learning is proposed in this paper. The main contributions of this work can be summarized as follows. (1) GAN is introduced for the expansion of the target domain dataset, and Resnet-50 is used to extract the fault signal features of both source and target domain data. (2) The domain adaptation regularization item in the training process of the deep Resnet-50 is introduced, the data distribution difference between the source domain and the target domain is reduced, and the construction of the deep transfer fault diagnosis model is completed.

The rest of this article is organized as follows. In Section 2, basic prerequisite knowledge has been introduced, which includes TL and GAN. In Section 3, the overall structure of the proposed fault diagnosis algorithm is described. In Section 4, the performance of the proposed algorithm and the experimental verification results on the test dataset are discussed. In Section 5, general conclusions are summarized.

## 2. Background Theories

### 2.1. Transfer Learning Description

**Figure 1:**
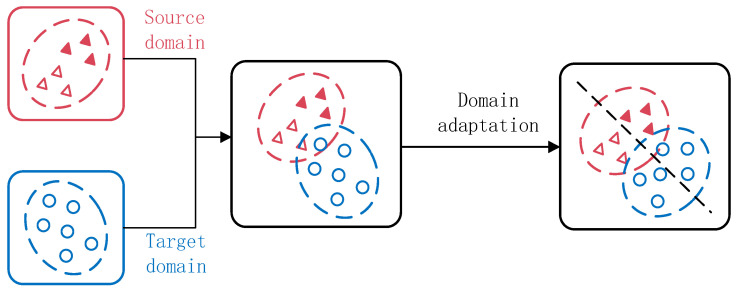
Schematic diagram of mechanical fault diagnosis based on transfer learning.

TL is a method of using existing knowledge to solve problems in different but related fields. Under variable conditions, TL plays a vital role. The operating conditions of mechanical equipment will change facing different fault diagnosis fields. The fault signal data distribution is different under various working conditions, which are defined as different domains. The case where the data are labeled is defined as the source domain $D_{s}={\left\{x_{i}^{s},y_{i}^{s}\right\}}_{i=1}^{n_{s}}$, which includes $n_{s}$ samples. $y_{s}\in \Upsilon$ is the label of sample $x_{i}^{s}$, Υ={1,2,⋯,k} is the label domain, which has k healthy states. The source domain sample $x_{i}^{s}$ belong to sample space $\chi^{s}$, and data obeys marginal probability distribution $P_{s}(\chi^{s})$. Data unlabeled conditions are defined as target domains $D_{t}={\left\{x_{i}^{t}\right\}}_{i=1}^{n_{t}}$, and contain $n_{t}$ samples to be classified. Target domain sample $x_{i}^{t}$ belongs to sample space $\chi^{t}$, and data obey marginal probability distribution $P_{t}(\chi^{t})$. Through the source domain fault diagnosis knowledge to assist the training of the target domain diagnosis model, the source domain and the target domain should satisfy the following relationship: The label space $\Upsilon^{s}$ of the source domain data needs to contain the label space of the target domain data $\Upsilon^{t}$: $\Upsilon^{t}\subseteq \Upsilon^{s}\subseteq \Upsilon$; the source and target domains have different data distribution, $P_{s}(\chi^{s})\neq P_{t}(\chi^{t})$, so the fault diagnosis model of the source domain cannot be directly applied to the fault diagnosis problem of the target domain, which may lead to misjudgment of the health status of the mechanical equipment. In view of the above-mentioned domain difference problem, as shown in Figure 1, domain adaptation, through the fault diagnosis knowledge of the source domain, assists the target domain to identify the health status of the equipment, which can solve the problem of fault diagnosis for unlabeled target domains. This paper aims to build a deep transfer diagnosis model, adapt the data distribution of mechanical equipment in the source domain and the target domain, and realize the reuse of fault diagnosis knowledge from the source domain to the target domain.

### 2.2. Generative Adversarial Network

**Figure 2:**
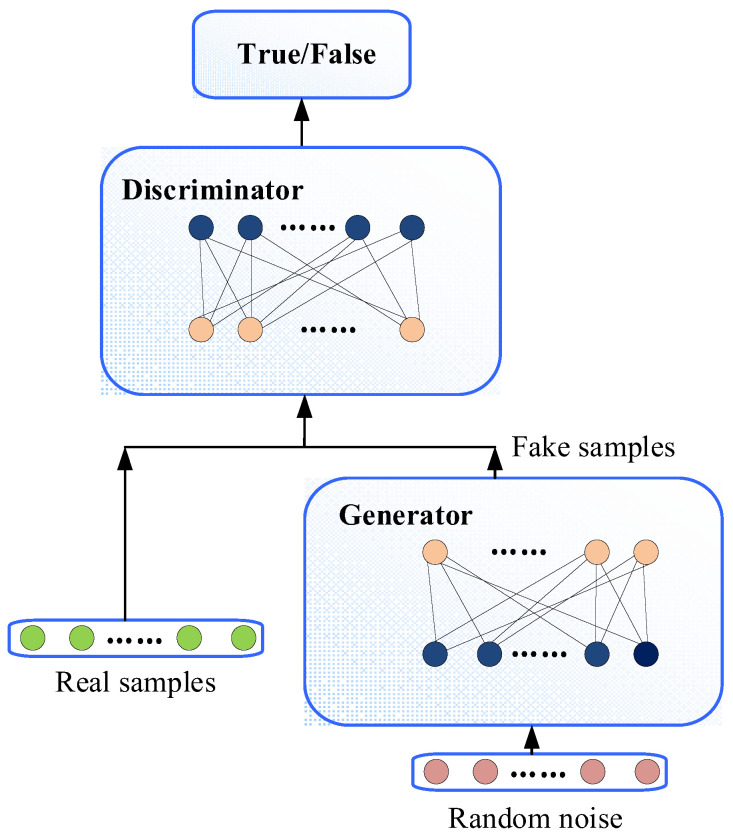
Schematic diagram of the basic process of GAN.

The GAN is a generative model based on adversarial theory. Specifically, the network contains two parts, i.e., a generator and a discriminator. The purpose of the generator is to learn the distribution law of real data samples and generate new data samples. The purpose of the discriminator is to determine whether the data come from the generator or the real data sample. These two pairs are continuously optimized in the confrontation and finally reach a balance. The basic composition framework of GAN is shown in Figure 2.

The input of the generator is random noise z, and the input of the discriminator is a sample of real data x and fake data generated by generator. Basically the optimization theory of GAN is confrontation training between the generator and discriminator. The generator continuously learns the data distribution of the sample, aiming to generate data that are infinitely close to the real sample, so that the discriminator will misjudge the generated false sample as a true sample; the discriminator aims to identify all true and false samples and make a correct decision. The two models conduct confrontation training and are optimized continuously. When the samples generated by the generator can be mixed, the spurious with the real, GAN network performance is optimal; that is, it has reached the Nash balance. The optimization process of GAN can be expressed as:

(1) $$\underset{G}{\min}\underset{D}{\max}V(D,G)=E_{x\sim P_{data}(x)}[\log D(x)]+E_{z\sim P_{z}(z)}[\log(1-D(G(z)))]$$

where V(D,G) is the objective function, G is the generator, D is the discriminator, x is the real sample data, $P_{data}$ is the data distribution of real samples, z is the random noise, and $P_{z}$ is the random noise data distribution.

The objective function V(D,G) can be divided into two parts: maximization Equation (2) and minimization Equation (3), which are the optimized objective functions of the generator and the discriminator, respectively.

(2) $$E_{x\sim P_{data}(x)}[\log D(x)]+E_{x\sim P_{g}(x)}[\log(1-D(x))]$$

(3) $$E_{x\sim P_{x}(x)}[\log(1-D(x))]$$

$P_{g}$ is the data distribution of fake samples generated by the generator. When the output of G is unchanged, the optimization of D can be calculated by Equation (4).

(4) $$D_{{}_{G}}^{*}(x)=\frac{P_{data}(x)}{P_{data}(x)+P_{g}(x)}$$

According to Equation (4), when $P_{data}=P_{g}$, $D_{G}^{*}(x)$ will obtain maximization.

GAN can be used for the extension of a fault signal dataset. Based on its advantages in feature extraction and distribution learning, GAN is considered for the extension of the target domain fault signal dataset to solve the problem of the scarcity of available data for mechanical equipment faults in practical engineering.

## 3. The Proposed Method

Aiming at the cross-condition problem of the scarcity of available data in fault diagnosis in the actual engineering environment and the low generalization ability of the single-condition model, a deep transfer fault diagnosis method is proposed. This method is composed of three parts, target domain data enhancement, transfer fault feature extraction, and distribution adaptation.

**Figure 3:**
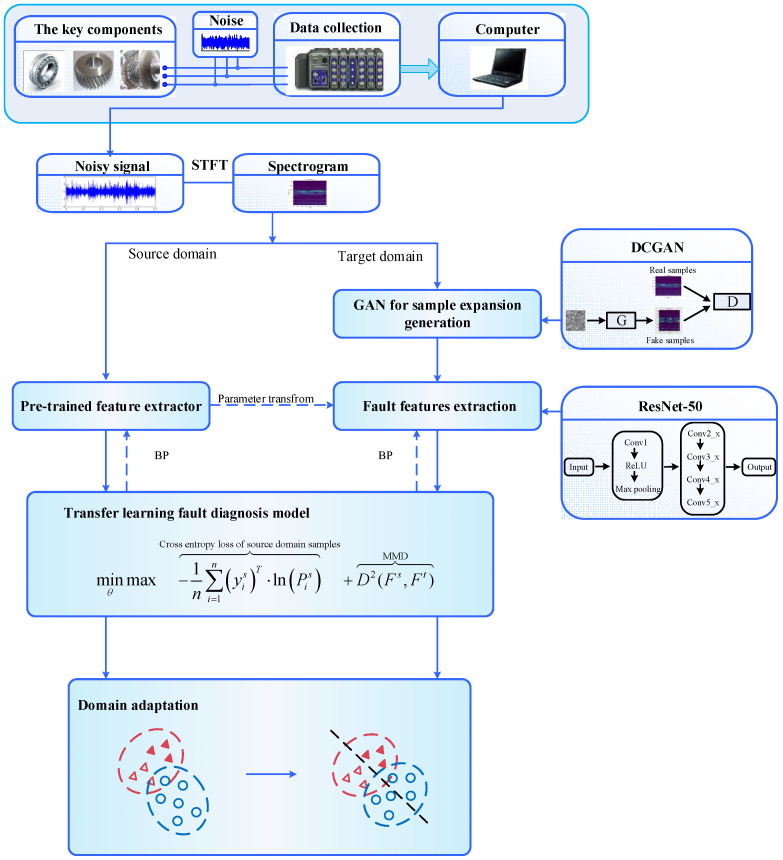
The entire flowchart of the proposed method.

Firstly, the measured one-dimensional fault vibration signal is converted into a two-dimensional image signal through the short-time Fourier transform (STFT). Once the dataset is established, a deep convolutional generative adversarial network (DCGAN) is used to extract the fault characteristic information of the target domain and generate similar fake samples to expand the target domain dataset. Then, transfer fault features in the source domain and target domain data are extracted by Resnet-50. Finally, the training process of Resnet-50 is constrained by domain adaptation regularization to form a deep transfer diagnosis model of mechanical faults, which reduces the distribution difference between the transfer fault features in the extracted source domain and target domain data. The fault diagnosis knowledge of the source domain equipment can be used to identify the health status of the target domain equipment. The overall block diagram of the proposed method is shown in Figure 3.

### 3.1. Deep Convolutional Generative Adversarial Network

**Figure 4:**
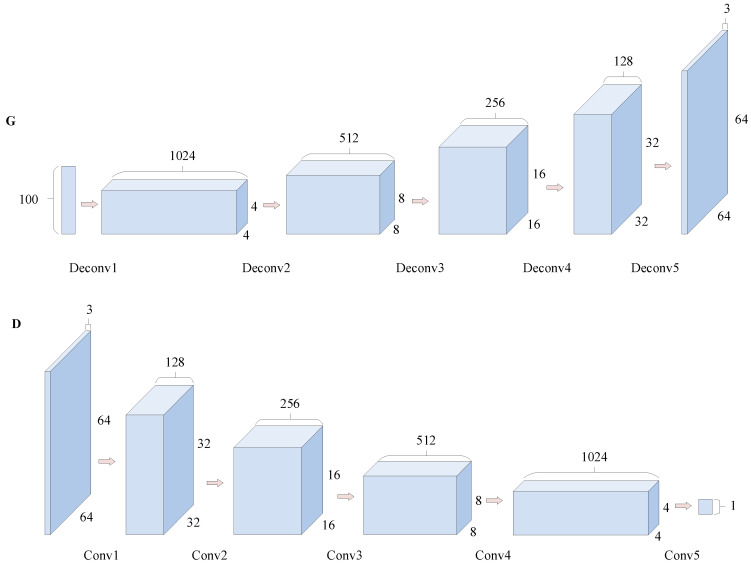
Schematic diagram of DCGAN. Here G denotes generator and D denotes discriminator.

The generator and discriminator of the original GAN are both composed of a multilayer perceptron (MLP), which cannot show better ability of image feature extraction. To make the extracted image feature more representative, a CNN is employed as a network model of the generator and discriminator, which is called DCGAN [33]. In DCGAN, the discriminator model is mainly composed of convolutional layers, and the generator model is mainly composed of transposed convolutional layers, as shown in Figure 4.

In the discriminator, the CNN is used to extract the fault features; in the generator, the transposed convolution structure is used to restore the information in the image, which is equivalent to the inverse operation of convolution.

DCGAN uses the powerful feature extraction capabilities of the CNN to model generators and discriminators. Compared with the original GAN using MLP for feature extraction, DCGAN extracts more features, and the extraction effect of image features is more prominent, which can more accurately restore the fault characteristics of the sample.

### 3.2. Resnet-50

In Figure 3, Resnet-50 is used to extract transfer fault diagnosis features of the source domain and target domain samples [34]. This model has strong feature expression ability and good generalization performance. Table 1 lists the structural information of Resnet-50.

In general, neural network models with complex structures have superior learning performance, and shallow neural networks have poor feature expression capabilities and generalization capabilities due to their simple model structure and are prone to underfitting. As the number of hidden layers increases, in the process of gradient back propagation, the continuous multiplication of gradients causes the training of deep neural network models to face the problem of gradient disappearance. The parameters of the model cannot be updated effectively, and the model finally shows poor prediction performance.

To deal with the problem of the disappearance of the gradient during the back propagation of the gradient in the deep neural network, a structure called identity mapping in Resnet-50 is processed. Specifically, the input of each residual block as a part of the output of the residual block can be directly used. This process can be expressed as:

(5) σ(x)=φ(x)+x

where x represents the input of the residual block, φ(x) represents the fault feature extracted from the residual block, and σ(x) represents the final output result of the residual block.

The derivation of the above formula with respect to the input gives:

(6) $$\sigma^{\prime }(x)=\varphi^{\prime }(x)+1$$

By repeatedly cascading multiple residual blocks, a network model with a deep structure can be built. Due to the structure of cascading multiple identity maps in the model, the chain derivation rule is adopted in the process of gradient backpropagation. In this way, the gradient of the deeper parameters in the model can be directly transferred to the previous layer. As a result, the parameters of each layer can be updated in time and the problem of gradient disappearance is well solved.

Therefore, Resnet-50 is used to process mechanical fault signals in this paper, which can establish a domain-sharing deep Resnet and extract transfer fault features in samples from the source and target domains. By adding domain adaptation regular terms, the difference in feature distribution between the source domain and the target domain is calculated, network parameters are optimized in backpropagation, and the health status of the samples in the source domain and the target domain is accurately classified.

### 3.3. Domain Adaptation Regularization Constraint

Maximum mean discrepancy (MMD) is a non-parametric distance indicator that measures the difference in the distribution of two datasets. After the monitoring sample data of the source domain and the target domain extract the deep fault features through Resnet, the distribution difference of the transfer fault features stays in the fully connected layer. Assuming that the transfer failure feature set is $F^{s}$, $F^{t}$, there is a reproducing kernel Hilbert space ℋ (RKHS), and there is a mapping function Φ(⋅)∈ℋ to map the transfer failure feature from the original feature space to the RKHS. The MMD value of the transfer fault characteristics of the source domain and the target domain can be defined as:

(7) $$D_{ℋ}(F^{s},F^{t})=\underset{\Phi \in ℋ}{\sup}\{E[\Phi (x_{i}^{s,F})]-E[\Phi (x_{i}^{t,F})]\}$$

where sup{⋅} is the set supremum, $x_{i}^{s,F}$ is the fault characteristics of the fully connected layer of the extracted source domain samples, and $x_{i}^{t,F}$ is the fault feature of the fully connected layer of the extracted target domain sample.

It can be seen from Equation (7) that there is always a mapping $\Phi^{*}(\cdot )$ in a complete RKHS so that the mean distance between the transfer fault characteristics of the source domain and the target domain reaches the minimum upper bound of the set. Based on the Gaussian kernel function to construct the RKHS, the empirical estimation of MMD can be expressed as [1]:

(8) $$D^{2}(F^{s},F^{t})=\frac{1}{n_{s}^{2}}\sum_{i=1}^{n_{s}}\sum_{j=1}^{n_{s}}k(x_{i}^{s},x_{j}^{s})-\frac{2}{n_{s}n_{t}}\sum_{i=1}^{n_{s}}\sum_{j=1}^{n_{t}}k(x_{i}^{s},x_{j}^{t})+\frac{1}{n_{t}^{2}}\sum_{i=1}^{n_{t}}\sum_{j=1}^{n_{t}}k(x_{i}^{t},x_{j}^{t})$$

where k(.,.) is the Gaussian kernel function.

By maximizing and minimizing the above function, the distribution difference between the extracted fault features of the source domain and the target domain can be reduced, and the fault diagnosis knowledge learned from the source domain can be reused in the diagnosis task of the target domain.

### 3.4. Training of Transfer Diagnosis Model

In this section, an intelligent diagnosis method for mechanical faults based on a generative confrontation network and deep TL is proposed, and we suggest that this work is a good start for a solution for cross-condition fault diagnosis with insufficient available data.

To improve the recognition accuracy of the target domain equipment health status and complete the transfer diagnosis task, combined with the domain adaptation regular term MMD, the following objective function for the transfer diagnosis model training is constructed:

(9) $$\underset{\theta }{\min}\max\overset{Cross\;\mathrm{entropy}\;\mathrm{loss}\;\mathrm{of}\;\mathrm{source}\;\mathrm{domain}\;\mathrm{samples}}{\overbrace{-\frac{1}{n}\sum_{i=1}^{n}{\left(y_{i}^{s}\right)}^{T}\cdot \ln\left(P_{i}^{s}\right)}}+\overset{\mathrm{MMD}}{\overbrace{D^{2}(F^{s},F^{t})}}$$

where θ is the parameter set to be trained for Resnet-50, $y_{i}^{s}$ is the binarization mark of source domain sample $x_{i}^{s}$, and $P_{i}^{s}$ is the probability distributions of $x_{i}^{s}$.

The optimization of the objective function can be summarized as the following steps:

(1) Collect one-dimensional fault vibration signals from mechanical equipment, segment the signals and perform short-time Fourier transform on them to convert them into two-dimensional fault characteristic spectrograms, and construct source and target domain datasets.
(2) Input the target domain data into DCGAN for training. Specifically, the fault feature information is extracted through the CNN in the discriminator, while the generator will transpose and convolve the input noise signal to restore the fault feature spectrogram. Meanwhile, the generator and the discriminator keep fighting against the training, and finally a fake sample similar to the target domain sample is obtained.
(3) Input the source domain and target domain samples into Resnet-50, extract the transfer fault features in the samples layer by layer, obtain the predicted label of the source domain sample and the pseudo label of the target domain, and combine the estimated value of MMD calculated by Equation (8), Furthermore, the objective function value of the transfer diagnosis model is calculated by Equation (9).
(4) Using the SGD optimization algorithm, the network parameters of Resnet-50 are updated layer by layer in the reverse direction. Return to step (3) until the output diagnostic accuracy rate reaches the termination condition.
(5) Save the diagnosis model network parameters, input the target domain test dataset, and the output result is the predicted probability distribution of the sample health label. The health label corresponding to the maximum value of the element in the probability distribution represents the predicted health status of the target domain sample.

## 4. Experiment Results

To verify the effectiveness of the proposed method, experiments were carried out on a Case Western Reserve University (CWRU) dataset and a Paderborn University (PU) dataset. The program of the formulated model was developed in python 3.6.7 with Pytorch deep learning library and implemented on Linux with an RTX 2080 Ti GPU.

### 4.1. CWRU Dataset

#### 4.1.1. Description of Data

**Figure 5:**
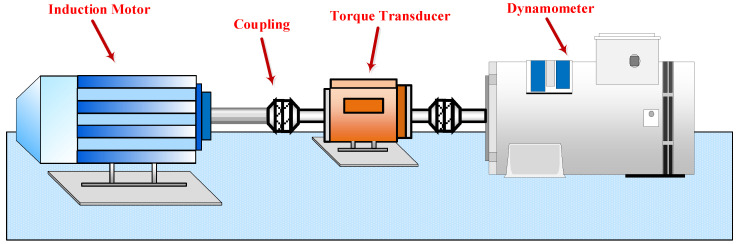
The experimental platform of CWRU.

The CWRU bearing dataset was collected from the experimental platform provided by Case Western Reserve University [35]. As shown in Figure 5, the experimental platform consists of an induction motor, coupling, torque transducer, and dynamometer. The data are divided into normal state and three failure states, namely, normal (NO), inner race fault (IF), outer race fault (OF), and roller fault (RF). At a 12 k sampling frequency, four load conditions (0 hp, 1 hp, 2 hp, 3 hp) were collected from the motor drive end of the vibration signal. When using electrical discharge machining (EDM), each fault state has three different fault size levels (i.e., 0.007 inch, 0.014 inch, 0.021 inch). Therefore, there are 10 kinds of bearing vibration data in different health states under each load, including one normal state and nine different positions and different size fault states.

**Figure 6:**
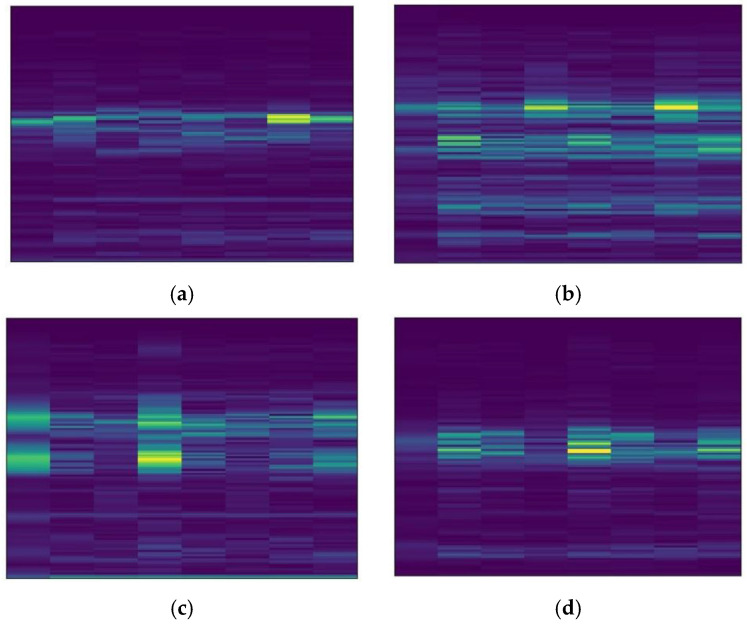
Spectrograms of 10 different label samples in CWRU domain A (**a**) NO, (**b**) IF07, (**c**) OF07, (**d**) RF07.

The datasets collected under four different load conditions are defined as domains A, B, C, and D, respectively. There are 2000 samples in each domain, including 10 different health states, 200 samples for each state, and each sample contains 1024 sampling points. The configuration information of the samples in each domain is shown in Table 2. The short-time Fourier transform is used to convert the one-dimensional vibration signal in the dataset into a two-dimensional spectrogram. Figure 6 shows the spectrogram of four different label samples in domain A.

#### 4.1.2. Deep Convolutional Generative Adversarial Network

DCGAN was used to extend the target domain dataset. Both the generator and discriminator use the Adam optimization algorithm and the learning rate is set to 0.0002. First, the discriminator is trained and the parameters of the generator are fixed so that the discriminator can judge the true sample as 1 and the false sample as 0. Then, the parameters of the discriminator are fixed and the generator is trained so that the discriminator judges the false sample generated by the generator as 1. The generator and the discriminator continue the adversarial training, update the parameters by the back propagation method, the epoch time of the network is set to 500, and finally the false samples with similar fault characteristics as the input samples are generated.

**Figure 7:**
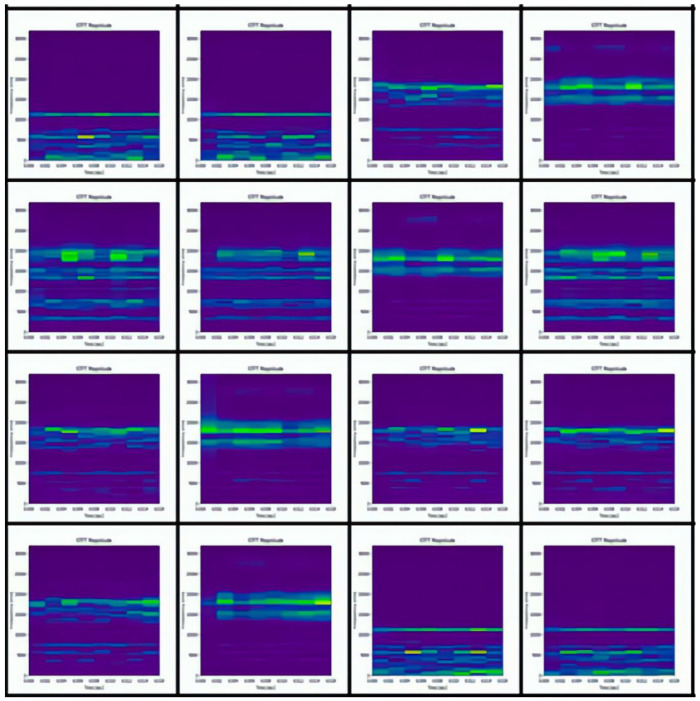
Part of fake samples in domain A generated by DCGAN.

The input of the network is 2000 samples of each domain, and data enhancement is performed on each domain separately. In particular, the label information is not used in the training process, and the output is an unlabeled fault feature spectrogram. Figure 7 shows some fake samples of domain A generated by DCGAN.

#### 4.1.3. Transfer Diagnosis Results

To evaluate the robustness of the proposed method more comprehensively, 12 transfer tasks were set: A→B, A→C, A→D, B→A, B→C, B→D, C→A, C→B, C→D, D→A, D→B, D→C. Taking A→B as an example, it means transfer from domain A to domain B. In other words, domain A is the source domain and domain B is the target domain. The source domain consists of 2000 labeled samples, and the target domain consists of 2000 unlabeled samples, including 1000 real samples and 1000 fake samples generated by DCGAN. The test set is the remaining 1000 labeled samples in the target domain. The purpose of the A→B transfer task is to use the fault diagnosis knowledge learned by the source domain (domain A) in the fault diagnosis task of the target domain (domain B) to assist the target domain in identifying the health status of the sample.

In the training process of the transfer diagnosis model, the main parameters of the proposed method are set as follows: use the SGD optimization algorithm to train the network with a dynamic learning rate. The learning rate is set to 0.01, dynamic attenuation. The batch size is set to 32 and the epoch is set to 100.

This experiment also used other methods for comparison to verify the superiority of the proposed method. All the methods involved in the experiment are listed as follows.

(1) CNN
(2) CNN + MMD
(3) Resnet-50

In contrast method CNN, CNN + MMD both use the same neural network model with seven layers. The structural information of the model is shown in Table 3. The classification accuracy is used to measure the diagnostic performance of the four models. The experimental results of the models are shown in Table 4.

Table 4 shows that the performance of the proposed method is better than the other models. Analyzing the experimental results in the table, the following conclusions can be summarized.

(1) It can be seen from Table 4 that the average accuracy of the proposed method reaches 98.5%, which is higher than other methods. DCGAN’s dataset expansion and domain adaptation enable the proposed method to learn more transfer fault characteristics.
(2) The traditional machine learning method CNN has the worst performance among all models because it does not consider how to reduce the distribution difference between the source domain and the target domain. The diagnosis accuracy of CNN + MMD has some improvement, which indicates that it is necessary to reduce the distribution difference between the source domain and the target domain when dealing with fault data under different working conditions. However, the diagnosis accuracy of CNN + MMD is lower than that of CNN in individual cases, such as the transfer task A→C, which indicates that the method cannot handle the fault diagnosis task under complicated and variable working conditions well, and the simple structure of CNN cannot effectively extract the common features in both the source domain and target domain.
(3) Resnet-50 has a higher diagnosis accuracy than the first two methods, which indicates its strong feature extraction capability. Compared with the proposed method, the difference in feature distribution between the source domain and target domain is not considered to be reduced in Resnet-50, so the diagnosis effect of Resnet-50 is still a little bit worse for the fault diagnosis tasks under different working conditions. The proposed method reduces the distribution difference between the two domains by minimizing the MMD and reduces the distance between the same labeled samples in the source domain and target domain to achieve better diagnosis results.

**Figure 8:**
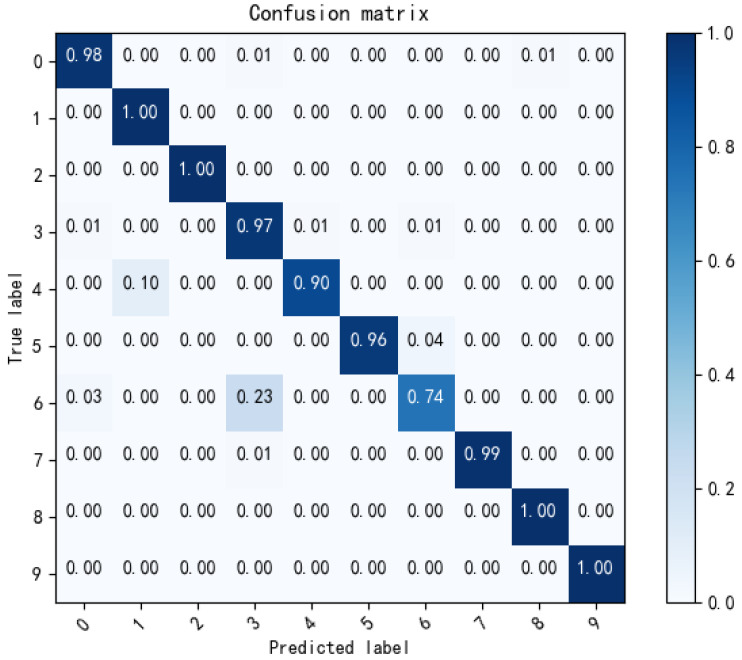
Confusion matrix of task D→A.

Feature visualization is used to further evaluate the superiority of the proposed method. The confusion matrix is used to display the diagnosis result of task D→A, as shown in Figure 8. It can be seen from Table 4 that task D→A has the lowest diagnosis accuracy among the twelve transfer tasks, and a total of 46 samples were misclassified. As shown in Figure 8, there are two main cases in which samples are misclassified. The first case is that the samples in category 4 are misclassified as category 1, both with 0.007 inch faults. The second case is that the samples in category 6 are misclassified as category 3, and they are both 0.021 inch faults. Table 4 shows that the diagnosis accuracy of task A→D is similar to that of task D→A, which is only 95.6%. Similarly, the misclassification is mainly due to the samples having the same fault size and the great difference in the working conditions between domain A and domain D.

**Figure 9:**
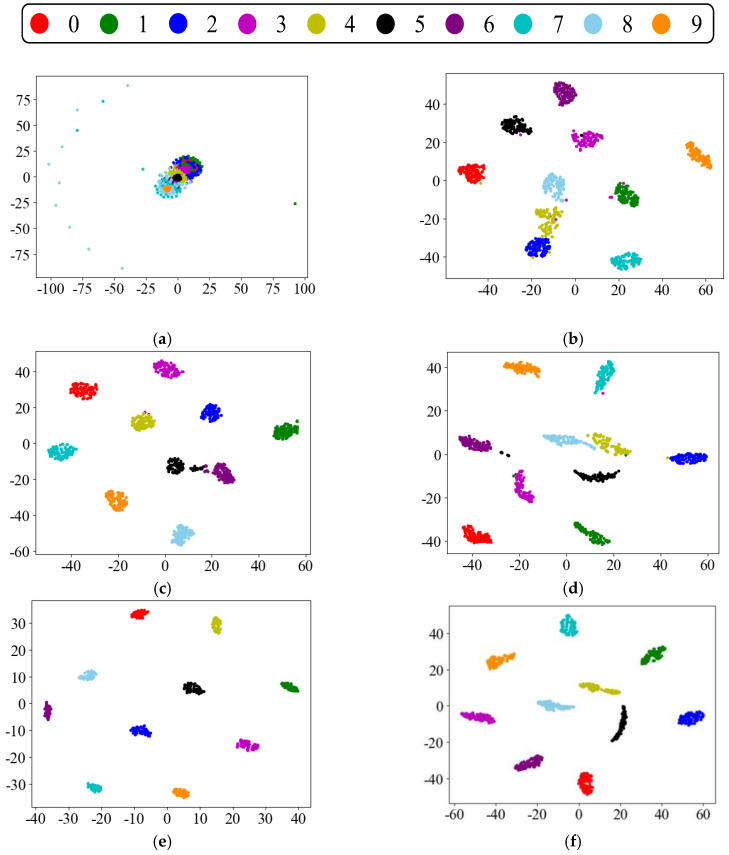
Visualization of the transferable features of task D→C using t-SNE. (**a**) Domain D original sample data; (**b**) CNN; (**c**) CNN + MMD; (**d**) Resnet-50; (**e**) proposed method-source domain; (**f**) proposed method-target domain.

In order to analyze the effect of the proposed method more visually, the t-distributed random neighborhood embedding (t-SNE) algorithm was introduced to reduce the dimensionality of the extracted fault features to a two-dimensional plane and present them in the form of a scatter plot. Taking task D→C as an example, by extracting the intermediate feature maps of several models and reducing the dimensionality by t-SNE, the visualization effect graph is shown in Figure 9. Most of the feature points in the original sample data of domain D are stacked together, and there is no clustering effect. The clustering effect of CNN performs the worst among all the models, and the inter-class distances of categories 2, 4, and 8 are small, and many feature points are misclassified. Resnet, which does not consider domain adaptation, suffers from the same problem. Although the accuracy has a small improvement over CNN, there is still the problem that some categories are relatively close to each other. In contrast, the CNN with the addition of the MMD domain adaptation regular term has better results in clustering, and the distribution of features in each class becomes more distinguishable. However, it is easy to confuse for faults of the same type but different sizes or different types with the same size, making it difficult for fault diagnosis methods to identify. The proposed method fully extracts the fault features by Resnet and calculates the feature distribution differences, thus reducing the offset between the source and target domains and effectively using the fault diagnosis knowledge learned in the source domain. From Figure 9e,f, it can be observed that the proposed method can clearly extract fault features and accurately identify the health status of the equipment, and each category can be well clustered.

### 4.2. PU Dataset

#### Description of Data

**Figure 10:**
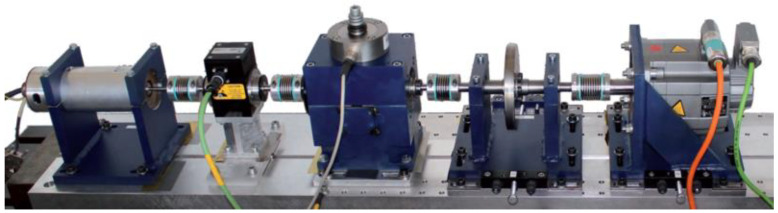
Schematic diagram of the bearing experiment system for obtaining PU dataset.

This subsection uses the Paderborn University (PU) dataset [36] to perform fault diagnosis classification experiments under variable operating conditions. The experimental rig consists of five parts, from left to right, a test motor, measurement shaft, bearing module, flywheel, and load motor, as shown in Figure 10. As shown in Table 5, the test bench first collects bearing failure data in a base setting, setting the rotational speed to n=1500 rpm, the load torque to M=0.7 Nm, and the radial force on the bearing to F=1000 N. Then the radial force is changed to F=400 N, the load torque is changed to M=0.1 Nm, and the bearing vibration signals are collected for the other two work conditions. Two sets of tests, artificial damage and real damage, were conducted on the inner and outer race of the bearing under each working condition. Therefore, there are nine different categories of vibration data in each working condition, including one normal state and eight fault states with different damage modes, different damage degrees, and different locations.

**Figure 11:**
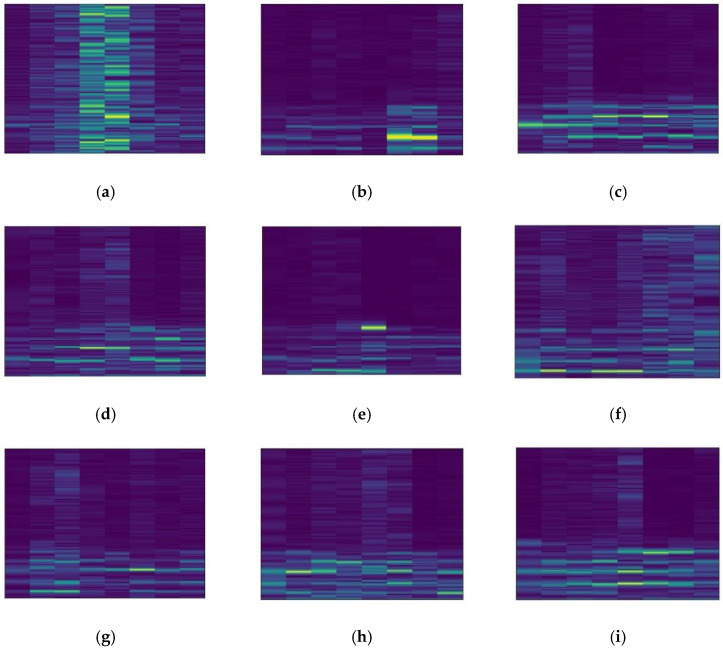
Spectrograms of 9 different labeled samples in domain A. (**a**) K001; (**b**) KA01; (**c**) KA03; (**d**) KA15; (**e**) KA16; (**f**) KI05; (**g**) KI07; (**h**) KI17; (**i**) KI18.

**Figure 12:**
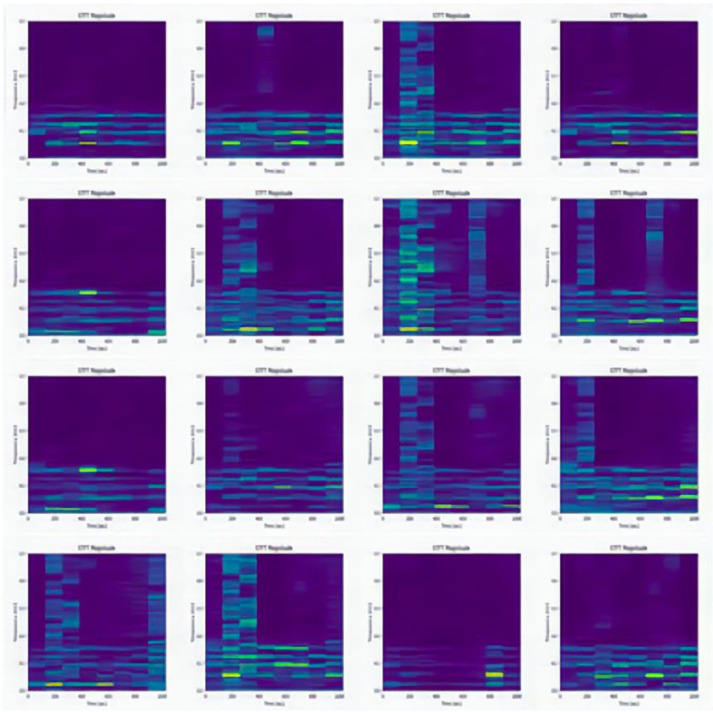
DCGAN generated partial domain A dummy sample.

The datasets collected under three different working conditions are defined as domains A, B, and C. There are 2250 samples in each domain, including sample data from nine different categories. There are 250 samples under each category, and each sample contains 1024 sampling points. The one-dimensional vibration signals in the dataset are converted into two-dimensional spectrograms by STFT to facilitate feature extraction by the diagnostic model. Figure 11 shows the spectrograms of the nine different labeled samples in domain A. Figure 12 shows some of the domain A dummy samples generated by DCGAN.

**Figure 13:**
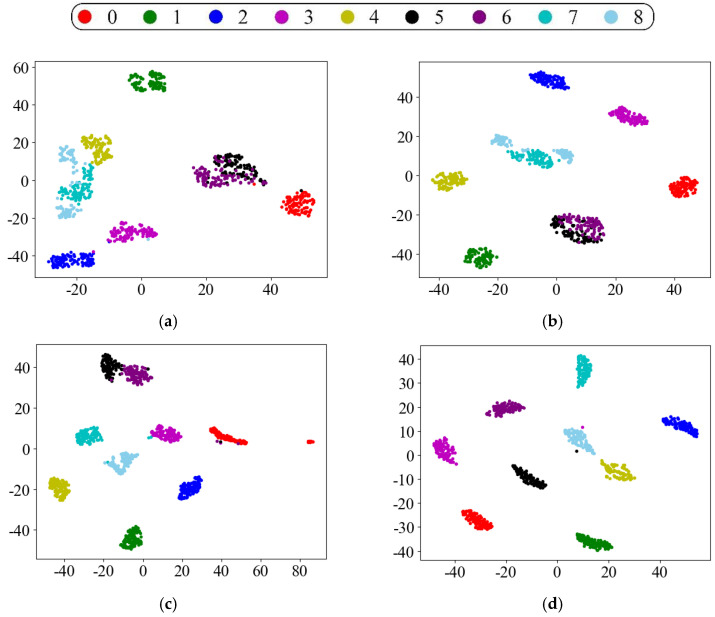
Visualization comparison of each method’s fault characteristics in Task C→A. (**a**) CNN; (**b**) CNN + MMD; (**c**) ResNet; (**d**) proposed method.

The experiments in this subsection also use four methods (CNN, CNN + MMD, ResNet, and the proposed method) to conduct comparison experiments to verify the effectiveness of the proposed method through six migration tasks. In each migration task, the source domain consists of 2250 labeled samples, and the target domain training set consists of 1250 real samples and 1000 fake samples, for a total of 2250 samples. The target domain test set consists of the remaining 1000 labeled samples. Again, the label information of the target domain is not used in the training process, but only in the calculation of the classification accuracy of the test set. The diagnostic accuracy of the four methods is shown in Table 6. Figure 13 shows the scatter plot of task C→A visualized by t-SNE features.

The comparative experimental analysis shows that the diagnostic accuracy of the proposed method is still better than other diagnostic models. The diagnostic accuracy of CNN is lower because it does not consider the domain feature differences, and the clustering effect of feature mapping is poor. With the addition of MMD for domain adaptation, the diagnostic accuracy of the model has been improved, and some fault states can be accurately identified. Since the network layers are shallow and do not learn deep fault features, there will still be some categories that are difficult to classify correctly, such as categories 5 and 6 and categories 7 and 8, which are all fault categories with the same damage location and the same damage type but different damage levels. Compared with the proposed method, ResNet does not add the domain adaptation regular term constraint, so there are still problems that the feature points of some categories are sticky and the inter-class distance is too small. Thus, the experimental validation in the PU dataset further illustrates the superiority and robustness of the proposed method for the task of bearing fault diagnosis under variable operating conditions.

## 5. Conclusions

Intelligent fault diagnosis plays an important role in improving the availability of mechanical equipment. Developing mechanical fault diagnosis models under different working conditions is the key to applying fault diagnosis technology in practice. This paper proposes a novel fault diagnosis model combining a generative confrontation network and TL to realize mechanical fault diagnosis under insufficient data volume and variable working conditions. The main contributions of the proposed model can be summarized as:

(1) Utilizing DCGAN to expand the target domain dataset to solve the problems of overfitting and negative transformation caused by insufficient data of the target domain dataset.
(2) Adopting the MMD regularization term to the feature extractor to minimize the difference in domain distribution.

Multiple sets of fault diagnosis experiments under variable work conditions were set up in two bearing fault datasets, respectively, to verify the effectiveness of the proposed method. The analyzed results demonstrate that the diagnostic accuracy of the proposed method is superior to other state-of-the-art fault diagnosis algorithms.

The intelligent fault diagnosis model proposed in this paper can still be improved. Specifically, MMD is only used as a strategy to measure distribution differences, and the metric is relatively singular. Future work will focus on adopting a variety of metrics to quantify the difference in feature distribution and formulating metrics with high computational efficiency. Future work will also focus on simplifying the formulated network models to improve computational efficiency. In addition, for the noisy data case, combining the sparsity-based denoising feature extraction method with the intelligent fault diagnosis model developed in this paper still remains to be conducted in future work.

## Figures and Tables

**Table 1:** Resnet-50 structure information table.

Layer Name	Output Size	Channels × Kernel Size
Input	3×224×224	-
Conv1	64×112×112	64×7×7, stride 2
BN, ReLU	64×112×112	-
Max pooling	64×56×56	64×3×3, stride 2
Residual block1: conv2_x	256×56×56	$\left[\begin{array}{l} 64\times 1\times 1\\ 64\times 3\times 3\\ 256\times 1\times 1 \end{array}\right]\times 3$
Residual block2: conv3_x	512×28×28	$\left[\begin{array}{l} 128\times 1\times 1\\ 128\times 3\times 3\\ 512\times 1\times 1 \end{array}\right]\times 4$
Residual block3: conv4_x	1024×14×14	$\left[\begin{array}{l} 256\times 1\times 1\\ 256\times 3\times 3\\ 1024\times 1\times 1 \end{array}\right]\times 6$
Residual block4: conv5_x	2048×7×7	$\left[\begin{array}{l} 512\times 1\times 1\\ 512\times 3\times 3\\ 2048\times 1\times 1 \end{array}\right]\times 3$
ReLU	2048×7×7	-
Average pooling	2048×1	2048×7×7
Fully connected: Softmax	10	-

**Table 2:** Sample configuration information table in each domain.

Bearing Fault Pattern	Diameters	Samples	Label	Abbreviation
Normal	-	200	0	NO
Inner race fault	0.007	200	1	IF07
	0.014	200	2	IF14
	0.021	200	3	IF21
Outer race fault	0.007	200	4	OF07
	0.014	200	5	OF14
	0.021	200	6	OF21
Roller fault	0.007	200	7	RF07
	0.014	200	8	RF14
	0.021	200	9	RF21

**Table 3:** Information of CNN structure.

Layer Name	Activation Function	Parameters
Input	-	-
Conv1	ReLU	Kernel_size = 3×3×16
Conv2	ReLU	Kernel_size = 3×3×32
Max pooling Conv3	- ReLU	Stride = 2 Kernel_size = 3×3×64
Conv4	ReLU	Kernel_size = 3×3×128
Adaptive MaxPool	-	Output_size = 4×4
Fully connected1	ReLU	4×4×128
Fully connected2	ReLU	1024
Fully connected3	ReLU	128
Output	-	10

**Table 4:** Diagnosis accuracy rate of 12 transfer tasks (%).

Transfer Task	CNN	CNN + MMD	Resnet	Ours
A→B	83.2	84.1	90.7	99.8
A→C	82.8	74.4	90.5	99.3
A→D	61.1	67.5	83.1	95.6
B→A	79.2	83.7	89.6	99.7
B→C	88.2	89.7	89.8	100.0
B→D	74.7	77.5	86.7	96.3
C→A	72.9	84.7	91.9	99.1
C→B	86.9	89.1	92.0	99.6
C→D	83.8	88.1	90.8	99.9
D→A	65.9	74.2	86.3	95.4
D→B	64.6	85.5	91.8	97.0
D→C	77.9	88.9	88.6	100.0
AVG	76.8	82.3	89.3	98.5

**Table 5:** Work conditions for obtaining PU dataset.

No.	Rotational Speed/rpm	Load Torque/Nm	Radial Force/N	Work Condition
A	1500	0.1	1000	N15_M01_F10
B	1500	0.7	400	N15_M07_F04
C	1500	0.7	1000	N15_M07_F10

**Table 6:** Classification accuracy of all methods (%).

Transfer Tasks	CNN	CNN + MMD	ResNet	Proposed Method
A→B	74.2	81.7	80.3	91.7
A→C	85.0	87.3	89.1	95.7
B→A	75.1	81.1	83.8	92.0
B→C	73.4	82.3	79.6	93.3
C→A	87.6	90.3	93.3	99.1

## Data Availability

This study did not report any data.
